# Invasive Meningococcal Disease in the Post–COVID-19 Era in South America

**DOI:** 10.3390/vaccines13111079

**Published:** 2025-10-22

**Authors:** Marco A. P. Sáfadi, Juan Francisco Falconi, Maria Gabriela Abalos, Lidia Serra, Angela Gentile, Alejandro Diaz, Claudia P. Cortes, Rodolfo Villena

**Affiliations:** 1 Department of Pediatrics, Santa Casa de São Paulo School of Medical Sciences, Rua Afonso Braz, 579, Cj 45, São Paulo 04511-011, Brazil; 2 Pfizer, Santiago 7560742, Chile; juanfrancisco.falconi@pfizer.com; 3 Pfizer Vaccines, Buenos Aires B1607EEV, Argentina; 4 Pfizer, Collegeville, PA 19426, USA; 5 Hospital de Niños Dr. Ricardo Gutiérrez, University of Buenos Aires, Buenos Aires C1425EFD, Argentina; 6 Hospital Pablo Tobón Uribe, Medellín 050015, Colombia; 7 Hospital General de Medellín Luz Castro de Gutiérrez ESE, Medellín 050015, Colombia; 8 Hospital Infantil Concejo de Medellín, Medellín 050015, Colombia; 9 Department of Internal Medicine, Universidad de Chile, Santiago 8330015, Chile; 10 Department of Pediatrics, Faculty of Medicine, Universidad de Chile, Infectious Disease Unit, Hospital de Niños Dr. Exequiel González Cortés, Santiago 8900000, Chile

**Keywords:** COVID-19, South America, disease incidence, meningococcal disease, *Neisseria meningitidis*

## Abstract

Background: During the COVID-19 pandemic, reductions in cases of bacterial diseases transmitted via the respiratory route were reported by the Invasive Respiratory Infection Surveillance Consortium. Here, we evaluate the epidemiology of invasive meningococcal disease (IMD) in Argentina, Brazil, Chile, and Colombia during and after the COVID-19 pandemic. Methods: The epidemiology of meningococcal disease was reviewed in selected South American countries through 2023 from publicly available national surveillance system databases. Results: The incidence of IMD decreased substantially in 2020 in Argentina, Brazil, Chile, and Colombia and was followed by a trend of increased disease. Similarly to observations in several European countries, the post-pandemic rebounds in cases of IMD in the four South American countries included in this analysis were mainly caused by serogroup B, that became one of the predominant serogroups causing IMD in all four countries. Conclusions: Enhanced surveillance of IMD, including genomic characterization of strains, is needed to inform public health policymakers and guide future vaccination strategies in the region.

## 1. Introduction

Invasive meningococcal disease (IMD), which is caused by the bacterium Neisseria meningitidis has resulted in substantial morbidity and mortality worldwide [1]. IMD typically presents as meningitis or sepsis with sudden disease onset, often progressing to severe disease in less than 24 h [2]. IMD case fatality rates have been estimated at 4% to 20%, and serious long-term sequelae including hearing loss, cognitive defects, visual abnormalities, and amputations are reported to occur in 10% to 20% of survivors [3,4,5]. IMD affects all age groups; the highest incidence rates are usually observed in infants, followed by young children and adolescents/young adults [6]. Recent evidence shows an increase in the prevalence and proportion of IMD cases among the elderly in high-income countries [7].

Globally, the epidemiology of IMD varies substantially by region and over time, largely driven by the sizable diversity and antigenic variability of N meningitidis [8,9,10]. N meningitidis is phenotypically and genetically categorized by serogroup and sequence type [8]. The most common disease-causing N meningitidis serogroups are A, B, C, W, and Y, with sporadic outbreaks of serogroup X in the meningitis belt of Africa [1,4]. Currently, serogroup B is the main cause of IMD in Europe, much of the Americas, and the Western Pacific [11,12]. Whole genome sequencing and multilocus sequence typing have led to the identification of closely related groups, termed clonal complexes, some of which belong to hyperinvasive or hypervirulent lineages that cause disease outbreaks worldwide [13]. These hypervirulent strains may be associated with higher frequencies of meningococcemia, worse clinical outcomes, and more atypical presentation of disease symptoms [13].

The most effective strategy to prevent IMD Is through vaccination. Vaccines for meningococcal disease that are currently in use globally include monovalent polysaccharide conjugate serogroup C (MenC) and serogroup A (MenA) vaccines, two protein-based vaccines for serogroup B (MenB), and numerous polysaccharide conjugate MenACWY vaccines, although national immunization programs (NIPs) from different countries include different vaccines [14,15]. Recently, a pentavalent conjugate vaccine targeting serogroups A, C, W, Y, and X was pre-qualified by the World Health Organization (WHO) for deployment in the African meningitis belt, and a pentavalent vaccine targeting the five most common serogroups (A, B, C, W, and Y) was approved by the US Food and Drug Administration [16,17,18].

During the COVID-19 pandemic, routine vaccine schedules around the world were disrupted [19,20,21]. In a survey conducted in eight countries (United States, United Kingdom, Italy, France, Germany, Argentina, Brazil, and Australia), approximately 50% of parents reported canceling or delaying a scheduled appointment for their children’s meningococcal vaccination during the pandemic [22]. The disruption of vaccination schedules, along with other COVID-19-related restrictions and containment measures, appear to have altered the transmission and incidence of diseases typically spread through the respiratory route [21,23]. In 30 countries and territories reporting to the Invasive Respiratory Infection Surveillance (IRIS) Consortium, there was a significant reduction in the risk of invasive disease caused by *Streptococcus pneumoniae*, *Haemophilus influenzae*, and *N*. *meningitidis* during the pandemic in 2020–2021 [21]. Furthermore, the reduced prevalence of respiratory viruses, particularly influenza, respiratory syncytial virus (RSV) and metapneumovirus, has been implicated as a factor that contributed to the reduction in the prevalence of invasive *S. pneumoniae* infections during the first 2 years of the pandemic [24]. In this regard, a better understanding of the correlation between the prevalence of these seasonal respiratory viruses and IMD rates still needs to be investigated. With the lifting of pandemic restrictions toward the end of 2021, IMD cases began to increase. During the first year of the pandemic in the Netherlands, all reported serogroup IMD cases (i.e., cases caused by serogroups B, C, W, and Y) decreased compared with the average number of serogroup-specific cases in the 5 years before COVID-19 [25]. In England, after COVID-19 containment measures were withdrawn, increased numbers of IMD cases caused by serogroup B were reported initially in adolescents/young adults, exceeding pre-pandemic levels, and later extending to other age groups [26,27]. This rebound in serogroup B IMD was also observed among adolescents and young adults in France and Australia, where MenB vaccination is only recommended for infants and toddlers. Interestingly, the number of IMD cases caused by serogroups C, W, and Y remained very low in countries with well-established Men ACWY vaccination programs (e.g., England and Australia), likely because of the protection afforded by the adolescent MenACWY conjugate vaccine [26,27]. Recently, it was reported that the overall incidence of IMD significantly decreased in Argentina, Brazil, Chile, and Uruguay in 2020, the first year of the COVID-19 pandemic [28].

An understanding of the trends In the epidemiology of IMD Is needed to help Inform public health policymakers and guide future vaccination strategies. Herein, we describe the evolving epidemiology of IMD during the COVID-19 pandemic and directly after the easement of restrictive measures across four South American countries—Argentina, Brazil, Chile, and Colombia—with available data on meningococcal disease.

## 2. Methods

The epidemiology of meningococcal disease (reported number of cases and/or incidence rates) was reviewed in selected South American countries through 2023, from publicly available national surveillance system databases. Data for Argentina were retrieved from SIREVA II (Surveillance Network System of Agents Responsible for Bacterial Pneumonia and Meningitis), a surveillance system implemented by the Pan American Health Organization and the Argentine Ministry of Health; data for Brazil were obtained from the Ministry of Health Brazil; data for Chile were obtained from the Chilean Institute of Public Health and the Department of Statistics and Health Information, Health Planning Division; and data for Colombia were retrieved from the Institute of National Health Columbia. Data extracted for each country included, where available, number of cases and incidence of meningococcal disease, disease distribution by age cohorts, serogroup-specific meningococcal disease, case fatality rates, and vaccination uptake. Literature searches were performed on an ad hoc basis to confirm the appropriate capture of all available case, incidence, and vaccination data.

## 3. Results

### 3.1. Argentina

SIREVA II reports the number of *N. meningitidis* isolates from patients with IMD. The reported number of *N. meningitidis* isolates decreased substantially from 2018 and 2019 (*n* = 64 and 63, respectively) to 2020 and 2021 (*n* = 17 and 14) and then increased in 2023 (*n* = 37; Table 1) [29]. Serogroup B, followed by serogroup W, accounted for the majority of isolates in 2018 through 2021, but the rebound in isolate numbers in 2023 was due predominantly to serogroup B (*n* = 23), followed by serogroup C (*n* = 11).

The Argentine Ministry of Health reported that the incidence of IMD decreased substantially from 2018/2019 to 2020/2021, followed by an increase in 2022 (Table 2) [30]. Across years 2018–2022, the IMD incidence per 100,000 inhabitants was highest in infants (range, ~0.5–3.7), followed by toddlers and young children (~0.25–0.7), and was lowest in all other age groups (<0.5). From 2018 onward the majority of IMD cases were caused by serogroup B, including IMD cases during the 2021 and 2022 rebound (Table 2). The case fatality rates for IMD in 2018, 2019, 2020, 2021, and 2022 were 15.6%, 9.3%, 3.3%, 15.0%, and 14.3%, respectively.

Argentina currently recommends MenACWY vaccination for infants and adolescents (Table S1) [30]. Since the start of the meningococcal vaccination program in 2017, vaccination rates in Argentina have trended upward in all age groups, except for the 11-year-old group, in which vaccination rates decreased substantially from 2018 to 2019 and then increased in 2020, 2021, and 2022 (Table S2) [30].

### 3.2. Brazil

**Figure 1:**
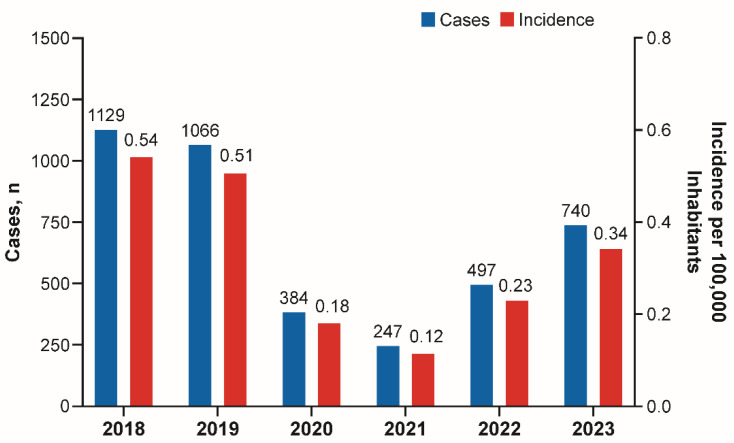
Invasive meningococcal disease cases and incidence rates in Brazil from 2018 to 2023 [31,32].

**Figure 2:**
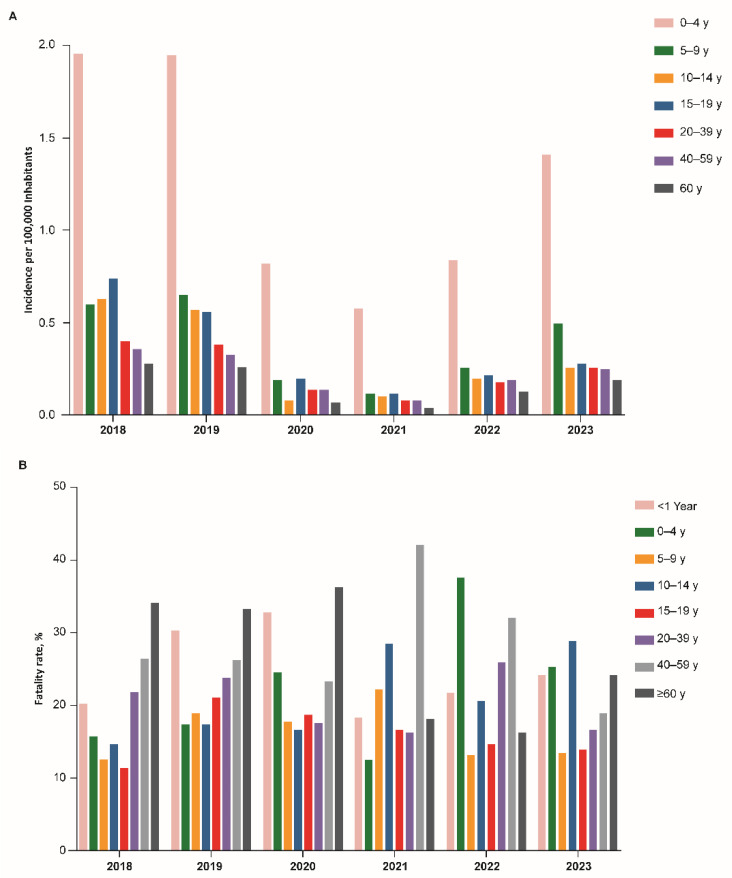

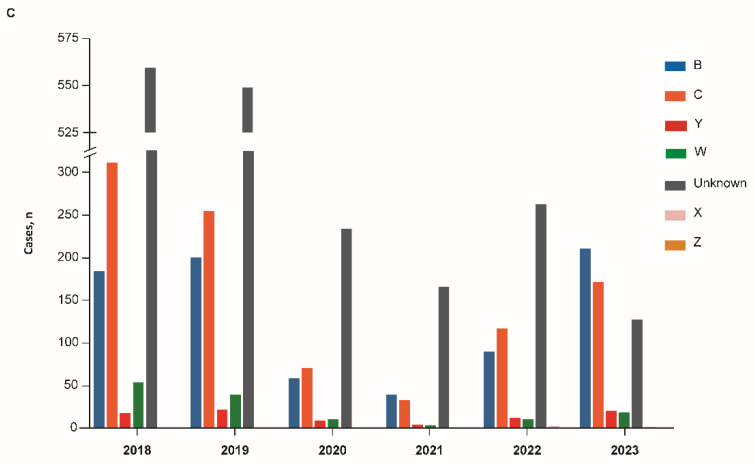
(**A**) Incidence of meningococcal disease by age group; (**B**) Fatality rate by age group; (**C**) Cases by serogroup in Brazil from 2018 to 2023 [31,32].

The Ministry of Health reported that meningococcal disease incidence rates (cases/100,000 inhabitants) declined substantially, from 0.54 (1129 cases) in 2018 and 0.51 (1066 cases) in 2019 to 0.18 (384 cases) in 2020 and 0.12 (247 cases) in 2021 (Figure 1) [31,32], which was the lowest incidence rate reported in Brazil since national surveillance was implemented [31,32]. However, in 2022, incidence rates were approximately two times higher (0.23 [497 cases]), and in 2023 approximately three times higher (0.34 [740 cases]) than in 2020 and 2021, showing a clear trend of increasing disease rates. Throughout the entire period, the highest incidence rates were consistently observed in children ≤4 years old (Figure 2A). Disease-associated overall case fatality rates tended to remain stable, with the highest case fatality rates reported in adults and elderly (Figure 2B).

In the first 2 years of the pandemic (i.e., 2020 and 2021), declines in incidence rates were observed for all serogroups, without significant changes in serogroup distribution (Figure 2C) [31,32]. However, in 2023 serogroup B became the predominant disease-causing serogroup, responsible for 49% of the cases with serogroups identified, followed by serogroup C (40%), serogroup Y (4.9%) and serogroup W (4.4%), with incidence rates of serogroup B exceeding the rates reported during the pre-COVID-19 period of 2018 and 2019. In 2022 and 2023, increased case numbers of meningococcal disease were observed for all prevalent serogroups (i.e., B, C, W, and Y).

In Brazil, vaccination with a MenC vaccine is recommended for infants and a MenACWY vaccine for adolescents (Table S1) [33]. In a survey of 223 parents in Brazil who had an appointment for meningococcal vaccination for their children, 8.5% reported canceling and 27.6% reported delaying their appointment because of the COVID-19 pandemic [34]. MenC vaccination coverage rates were severely affected in Brazil during the first years of the pandemic (Figure S1A) [35]. From 2022, a slight recovery was observed for vaccination with MenC whereas coverage with the MenACWY vaccine decreased in 2022 (Figure S1).

### 3.3. Chile

**Figure 3:**
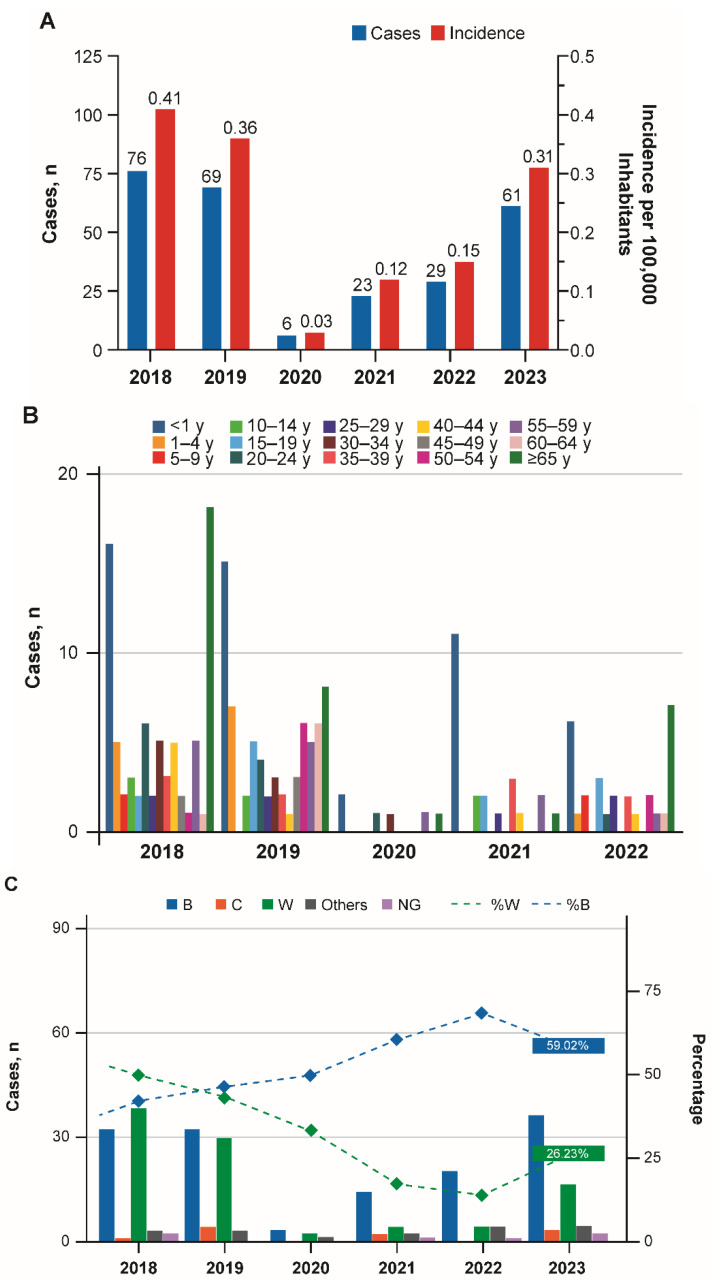
(**A**) Number of cases and incidence rates of invasive meningococcal disease in Chile; (**B**) Cases by age group in Chile; (**C**) Cases by *Neisseria meningitidis* serogroup in Chile from 2018 to 2023 [36,37,38,39]. NG, not groupable. Others include serogroups Y and Z.

Data from the Ministry of Health reported that IMD incidence rates (cases/100,000 inhabitants) in Chile decreased substantially from 0.41 in 2018 and 0.36 in 2019 to a low of 0.03 in 2020 (Figure 3A) [36,37]. Incidence rates (cases/100,000 inhabitants) increased in 2021 (0.12), 2022 (0.15), and 2023 (0.31) [38]. Declines in IMD in 2020 and 2021 occurred across all age groups (Figure 3B) [38]. Cases predominantly occurred in the infant and toddler age groups and in those ≥65 years of age before (2018–2019) and after (2022–2023) the COVID-19 pandemic; during the pandemic (2020–2021) cases predominantly occurred in infants (Figure 3B; Table S3) [39]. The decline in cases of IMD in 2020 occurred across all serogroups, whereas the rebound in numbers of cases of IMD in 2021 and 2022 was almost entirely caused by serogroup B (Figure 3C) [38]. In 2023, significant increases occurred in serogroup W cases among adults as well as serogroup B cases among infants and children aged ≤4 years.

In Chile, MenB vaccination with two primary doses, starting at 2 months of age, and a booster dose has been recommended for toddlers since 2023, and a MenACWY vaccine is recommended for those 12 months of age (Table S1) [40]. Vaccination uptake rates for the MenACWY vaccine in the target population were 94.2%, 96.2%, 88.7%, 89.6%, 95.7%, and 92.8% in 2018, 2019, 2020, 2021, 2022, and 2023, respectively (Figure S2A) [41]. MenB vaccination uptake was 98.7% (dose 1) and 96.5% (dose 2) in 2023 (Figure S2B) [41].

### 3.4. Colombia

**Figure 4:**
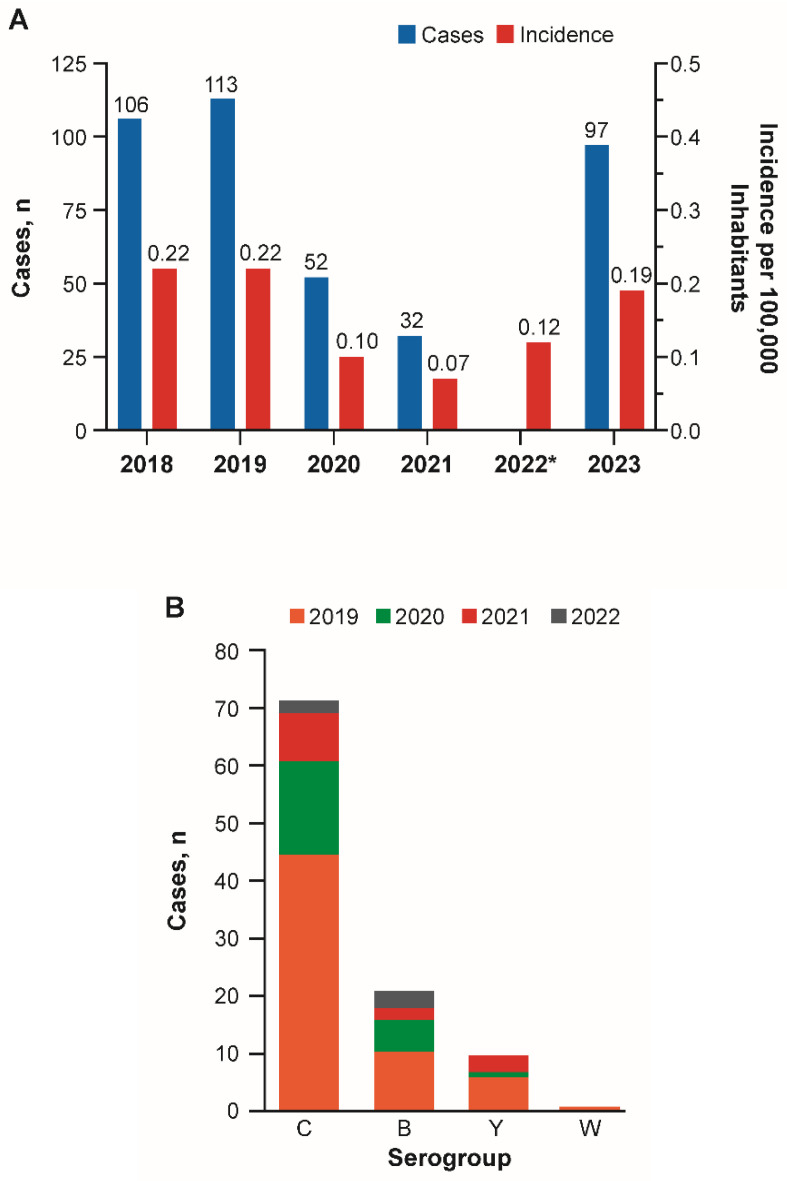
(**A**) Incidence and number of cases for meningococcal disease from 2018 to 2023 in Colombia; (**B**) Number of serogroup isolates from 2019 through the first half of 2022 in Colombia [42]. * Data regarding number of cases not available.

Data from Colombia’s Institute of National Health reported that meningococcal disease incidence rates per 100,000 declined substantially from 0.22 in 2018 and 2019 to 0.10 in 2020 and 0.07 in 2021, followed by increases to 0.12 in 2022 and 0.19 in 2023 (Figure 4A) [42]. From 2019 through the first half of 2022, most IMD cases were caused by serogroups C, B, and Y (Figure 4B) [42]. The number of C, B, and Y isolates decreased substantially from 2019 through 2021 (Figure 4B). Most cases of IMD from 2019 through 2021 were caused by serogroup C; serogroup B became the most prevalent serogroup during the first half of 2022 (Figure 4B). In 2023, 13 serogroup B cases, 16 serogroup C cases, 24 serogroup Y cases, and two serogroup W cases were reported by the Public Health Surveillance System [43].

## 4. Discussion

A trend in significant decreases in IMD during the COVID-19 pandemic, coincident with pandemic-related restrictions, followed by a slower rebound in disease rates coincident with lifting of restrictions, has been observed across 30 countries and territories in the IRIS Consortium [21]. In early 2020, the WHO declared that Latin America was the epicenter of the COVID-19 pandemic [44]. Accordingly, many measures were implemented across Latin American countries to limit social contact and prevent COVID-19 spreading, such as the use of face masks, curfews, quarantines, and non-presence in schools and workplaces [44]. As reported in other countries, such restrictions likely limited the spread of other communicable diseases, including influenza and RSV.

It was recently reported that the overall IMD incidence significantly decreased in Argentina, Brazil, Chile, and Uruguay in 2020, the first year of the COVID-19 pandemic [28]. In this evaluation of publicly available data during and after the pandemic across four South American countries (Argentina, Brazil, Chile, and Columbia), we found that there was a large decline in meningococcal disease incidence in 2020 followed by a rebound in disease rates in 2021 in Chile, as was previously reported [28], and in 2022 in Argentina, Brazil, and Colombia. In Brazil and Colombia, where serogroup C was the most prevalent serogroup before the pandemic, rebounds in IMD were predominantly caused by serogroup B in Columbia and serogroups C and B in Brazil, with serogroup B becoming the most predominant serogroup in Brazil in 2023. In Colombia, where serogroup C was the most prevalent serogroup before the pandemic, the rebound in IMD in 2022 was predominantly caused by serogroup B, whereas in Brazil, a similar proportion of cases caused by serogroups B and C, the predominant serogroups causing IMD in Brazil, were reported in 2022 compared with the period before the pandemic. In Argentina and Chile, where most IMD cases were caused by serogroups B and W before the pandemic, the rebound in IMD in 2022 was mostly caused by serogroup B.

Data regarding the most affected age groups were available from Argentina, Brazil, and Chile. Across all three countries, the highest incidences of IMD were typically observed in infants both before the pandemic and during the post-pandemic rebound. In Argentina, a secondary peak in incidence was seen among toddlers and young children, and in Chile, secondary peaks were seen among older adults. These patterns contrast with epidemiological trends in Europe, the United States, and Australia, where IMD—and particularly serogroup B IMD—secondarily impacts adolescents and young adults [27,45]. Differences in age-specific IMD incidence between global regions is consistent with observed differences in age-specific meningococcal carriage rates; adolescents and young adults are the primary carriers in Europe and other areas where serogroups B and C predominate, whereas these trends may differ in areas where serogroup A predominates, including Africa, Asia, and Russia [46]. Very limited data are available regarding meningococcal carriage by age group in Latin and South America.

In the past 15 years, three South American countries have implemented meningococcal conjugate vaccines (MenC and/or MenACWY) into their NIPs, resulting in a reduced burden of meningococcal disease [28]. As the result of a significant increase in MenW disease in Chile in 2011–2012, a catch-up vaccination program with a conjugated MenACWY vaccine was initiated in children 9 months to 5 years old [47]. MenACWY was introduced into the NIP as a single dose for toddlers in Chile in 2014 and for infants, toddlers, and adolescents in Argentina in 2017 [28]. MenB vaccination for children aged 2, 4, and 18 months was introduced in Chile’s NIP during the second semester of 2023 [28]. Given the recent emergence of serogroup B as the predominant cause of IMD nationwide, MenB vaccination is likely to substantially decrease IMD incidence among age groups targeted by the vaccination program over the coming years. The MenACWY vaccine was also introduced in Brazil’s NIP for adolescents in 2020, although a MenC vaccine had been previously introduced in 2010 for infants and 2017 for adolescents. In 2020, worldwide routine vaccinations declined because of vaccination programs being interrupted, temporarily suspended, or delayed [48]. Such decreases in vaccination rates are of serious concern for controlling vaccine-preventable diseases such as meningococcal disease. Although a clear reduction in rates of vaccination was observed in Brazil during the first 2 years of the pandemic, in Argentina and Chile the rates of vaccination did not decrease or only slightly decreased during and after the pandemic. Despite this, there was an increasing trend in the incidence of IMD in the post-pandemic years across all four countries assessed in this study, with the common finding that serogroup B was the predominant serogroup associated with these rebounds. Although decreases in vaccination likely resulted in increased disease incidence, the lockdown measures taken during the pandemic, such as social distancing, quarantining, and mandatory mask use, may have also contributed to this increase by reducing exposure to circulating microorganisms, including meningococcal hyperinvasive isolates, causing a relative lack of immune stimulation and creating an “immunity debt” [49], leaving increased numbers of people more susceptible to microbial diseases such as IMD.

Interestingly, a common finding in all four of the countries studied here was the increased trend in incidence rates of serogroup B in 2022, a phenomenon similar to one reported in the United Kingdom and other European countries [50]. This may reflect the lack of vaccination programs targeting this serogroup during that period, unlike serogroups A/C/W/Y which are targeted by current MenACWY programs implemented in Argentina, Brazil, and Chile. In this regard, Chile has recently announced that an infant MenB vaccination program has been implemented. In the future, availability of pentavalent vaccines targeting serogroups A, B, C, W, and Y [16,17] in Latin and South American regions could also help to combat the rise in serogroup B disease while simplifying vaccination schedules.

How high the rebound in IMD incidence will be in the upcoming years in South America is unknown. Brazil and Argentina, by maintaining adolescent immunization programs with the quadrivalent MenACWY conjugate vaccine, are better positioned to mitigate any re-emergence of disease caused by serogroups A, C, W, and Y. This adolescent strategy is expected to sustain herd and direct protection in the age groups that drive transmission. In contrast, Chile, without a comparable ACWY program in adolescents, appears in principle to be more vulnerable to a resurgence of ACWY disease, and particularly serogroup W disease, should transmission pressures rise. Of course, the actual risk will also depend on local epidemiology, vaccine coverage across other age cohorts, and the timeliness of surveillance and outbreak responses.

Many countries throughout this region continue to face enormous barriers to healthcare in part because of income disparities, where in some cases up to two thirds of the population has reported not seeking medical care when sick [51,52,53]. Under these circumstances, it is likely that even fewer individuals would seek medical care when healthy, such as to receive a vaccine. National and international organizations continue to prioritize these issues, and reforms designed to guarantee equitable healthcare access have made an impact in some countries such as Paraguay, Columbia, Peru, and Ecuador [51,53]; however, much more work is needed to address the complex and multifactorial socioeconomic inequalities impacting the region [52,53]. Regarding IMD specifically, the increasing incidence occurring after the pandemic further highlights the importance of maintaining or expanding the meningococcal vaccination programs, particularly targeting predominant serogroups, to promote equitable access that translates directly to improved health outcomes across the socioeconomic spectrum.

Strengths of this review included the quality of the data sources, which were national surveillance databases, and the inclusion of both IMD incidence and vaccination data to allow for contextualization of changes in disease rates over time. However, all results and interpretations are subject to the potential limitations of country-specific data collection methods (e.g., underreporting in rural areas) and are also difficult to compare across countries because of inconsistencies in reporting standards and serogroup testing methods.

## 5. Conclusions

The incidence of IMD decreased substantially in 2020 throughout Argentina, Brazil, Chile, and Colombia and was followed by a trend of increased disease, mainly associated with serogroup B, in the post-COVID-19 pandemic years. Continued surveillance of IMD cases, vaccination uptake, and genomic strain characterization will be important for evaluating the effectiveness of meningococcal disease prevention strategies and optimizing public health policies.

## Figures and Tables

**Table 1:** Meningococcal disease isolates reported by SIREVA II in Argentina in 2018–2021 and 2023 [29].

Year	Total Cases, *n*	Serogroup, *n*				NG	Other Groups, *n*
		B	C	W	Y		
2018	64	34	7	22	0	1	-
2019	63	29	9	20	3	0	2
2020	17	12	0	3	1	1	-
2021	14	8	2	4	0	0	-
2023	37	23	11	1	2	0	-

NG = nongroupable; SIREVA = Surveillance Network System of Agents Responsible for Bacterial Pneumonia and Meningitis.

**Table 2:** Invasive meningococcal disease case numbers, incidence rates, and percentage distribution of serogroups in Argentina from 2018 to 2022 [30].

Year	Total Cases, *n*	Incidence *^,†^	Serogroup, %*				
			B	C	W	Y	Other
2018	96	0.22	59	9	31	0	1
2019	97	0.22	51	14	27	4	2
2020	30	0.06	78	0	17	6	0
2021	20	0.03	47	30	24	0	0
2022	56	0.13	65	12	15	6	4

* Values are approximate. **^†^** Incidence per 100,000 inhabitants.

## Data Availability

This article is based on the published literature and therefore does not contain any applicable data sets.

## Supplementary Materials

The following supporting information can be downloaded at: https://www.mdpi.com/article/10.3390/vaccines13111079/s1, **Table S1:** Summary of current meningococcal vaccine schedules in four South American countries. **Table S2:** Vaccination coverage against meningococcus (serogroups A, C, W, and Y) in Argentina from 2018 to 2022*. **Table S3:** Meningococcal disease cases by age group in Chile, 2023. **Figure S1:** Vaccination coverage against MenC (A) and MenACWY (B) in Brazil from 2018 to 2022. **Figure S2:** Vaccination coverage against MenACWY (A) and MenB (B) in Chile from 2018–2023. [30,35,38,40,41,54,55]

## Abbreviations

IMD	Invasive meningococcal disease
IRIS	Invasive Respiratory Infection Surveillance
MenA	Monovalent polysaccharide conjugate serogroup A vaccine
MenACWY	Polysaccharide conjugate serogroups A, C, W, and Y vaccine
MenB	Protein-based serogroup B vaccine
MenC	Monovalent polysaccharide conjugate serogroup C vaccine
NIP	National immunization program
RSV	Respiratory syncytial virus
SIREVA II	Surveillance Network System of Agents Responsible for Bacterial Pneumonia and Meningitis
WHO	World Health Organization
