# Supplementary material for: Invasive Meningococcal Disease in the Post–COVID-19 Era in South America
Source: Vaccines (Basel). 2025 Oct 22;13(11):1079. doi: 10.3390/vaccines13111079 (PMC12656551; doi:10.3390/vaccines13111079)
Supplement: Supplementary file 1 [file vaccines-13-01079-s001.zip › vaccines-3837401_References.pdf]

REFERENCES

1. Ministry of Health Argentina. National Epidemiological Bulletin. Available online: https://www.argentina.gob.ar/salud/boletin-epidemiologico-nacional/boletines-2024 (accessed July 1, 2024).

2. Ministry of Health of Brazil. Programa Nacional de Imunizações. Available online: https://www.gov.br/saude/pt-br/vacinacao/calendario (accessed May 6, 2024).

3. Instituto de Salud Pública de Chile. Programa Nacional de Immunizaciones. Available online: https://www.minsal.cl/programa-nacional-de-inmunizaciones/ (accessed July 1, 2024).

4. Coronell-Rodriguez, W.; Caceres, D.C.; Cintra, O.; Guzman-Holst, A. Epidemiology of Invasive Meningococcal Disease in Colombia: A Retrospective Surveillance Database Analysis. *Infect Dis Ther* **2023**, *12*, 2709-2724; https://dx.doi.org/10.1007/s40121-023-00886-y.

5. Instituto de Salud Pública de Chile. Informe de Resultados de Vigilancia de Laboratorio Enfermedad Invasora *Neisseria meningitidis*. Chile, 2023. Available online: https://www.ispch.cl/wp-content/uploads/2024/03/Informe-Neisseria-meningitidis-SE-1-52-2023_08febrero2024.pdf (accessed June 6, 2024).

6. Ministry of Health Brazil. Immunizations - Coverage - Brazil. Available online: http://tabnet.datasus.gov.br/cgi/dhdat.exe?bd_pni/cpnibr.def (accessed September 30, 2024).

7. Ministry of Health of Chile. Preliminary coverages. Available online: https://vacunas.minsal.cl/coberturas-preliminares/ (accessed September 27, 2024).
