# Supplementary material for: Invasive Meningococcal Disease in the Post–COVID-19 Era in South America
Source: Vaccines (Basel). 2025 Oct 22;13(11):1079. doi: 10.3390/vaccines13111079 (PMC12656551; doi:10.3390/vaccines13111079)
Supplement: Supplementary file 1 [file vaccines-13-01079-s001.zip › vaccines-3837401_Supplementary Table S1.pdf]

**Supplementary Table S1. Summary of current meningococcal vaccine schedules in four South American countries.**

| Country | Vaccine program |
|---|---|
| Argentina [1] | MenACWY – In infants, primary doses at 3 and 5 months of age followed by a booster at 15 months of age. In adolescents, a single dose at 11 years of age. |
| Brazil [2] | MenC/MenACWY – In infants, doses of MenC at 3 and 5 months of age with a booster dose at 12–15 months of age. A single dose of MenACWY in adolescents 11–14 years of age. |
| Chile [3] | MenACWY – Recommended at 12 months of age.<br>MenB – Recommended for infants at 2 and 4 months of age with a booster dose at 18 months of age. |
| Colombia [4] | No meningococcal vaccines are recommended in routine schedules. Both MenACWY and MenB are available for the private market and recommended for outbreak control. |

MenACWY, meningococcal serogroups ACWY conjugated vaccine; MenB, meningococcal serogroup B vaccine; MenC, meningococcal serogroup C conjugated vaccine.
