# Supplementary material for: Invasive Meningococcal Disease in the Post–COVID-19 Era in South America
Source: Vaccines (Basel). 2025 Oct 22;13(11):1079. doi: 10.3390/vaccines13111079 (PMC12656551; doi:10.3390/vaccines13111079)
Supplement: Supplementary file 1 [file vaccines-13-01079-s001.zip › vaccines-3837401_Supplementary Table S2.pdf]

Supplementary Table S2. Vaccination coverage against meningococcus (serogroups A, C, W, and Y) in Argentina from 2018–2022* [1].

Year	3 months, %	5 months, %	15 months (booster), %	11 years, %
2018	77	66	44	37
2019	75	62	46	23
2020	78	69	62	55
2021	83	79	78	68
2022†	86	81	77	73

*Values are approximate.

†Data reported as of June 2023.
