# Supplementary material for: Invasive Meningococcal Disease in the Post–COVID-19 Era in South America
Source: Vaccines (Basel). 2025 Oct 22;13(11):1079. doi: 10.3390/vaccines13111079 (PMC12656551; doi:10.3390/vaccines13111079)
Supplement: Supplementary file 1 [file vaccines-13-01079-s001.zip › vaccines-3837401_Supplementary Table S3.pdf]

Supplementary Table S3. Meningococcal disease cases by age group in Chile, 2023 [5].

Age group, years	Total cases, n
<1	14
1–4	8
5–9	1
10–14	1
15–19	2
20–24	4
25–29	2
30–34	2
35–39	1
40–44	7
45–49	1
50–54	4
55–59	3
60–64	5
≥65	6
Total	61
