# Supplementary figures and images for: Invasive Meningococcal Disease in the Post–COVID-19 Era in South America
Source: Vaccines (Basel). 2025 Oct 22;13(11):1079. doi: 10.3390/vaccines13111079 (PMC12656551; doi:10.3390/vaccines13111079)

**Supplementary Figure S1. (A) Vaccination coverage against MenC; (B) MenACWY in Brazil from 2018–2022 [6].**

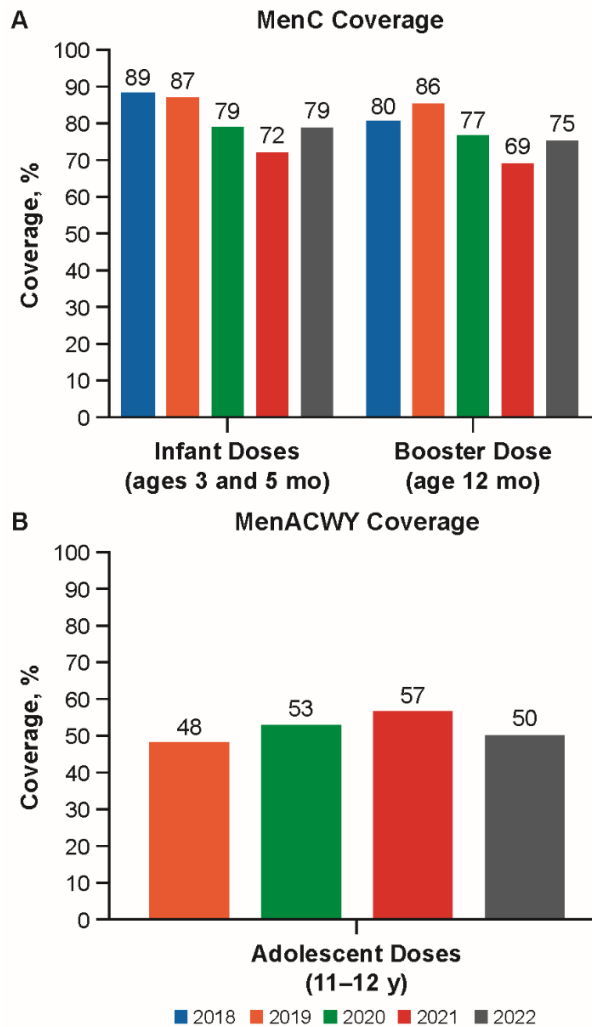

Supplement: Supplementary file 1 [file vaccines-13-01079-s001.zip › vaccines-3837401_Supplementary Figure S1.pdf]

**Supplementary Figure S2. (A) Vaccination coverage against MenACWY; (B) MenB in Chile from 2018–2023[7].**

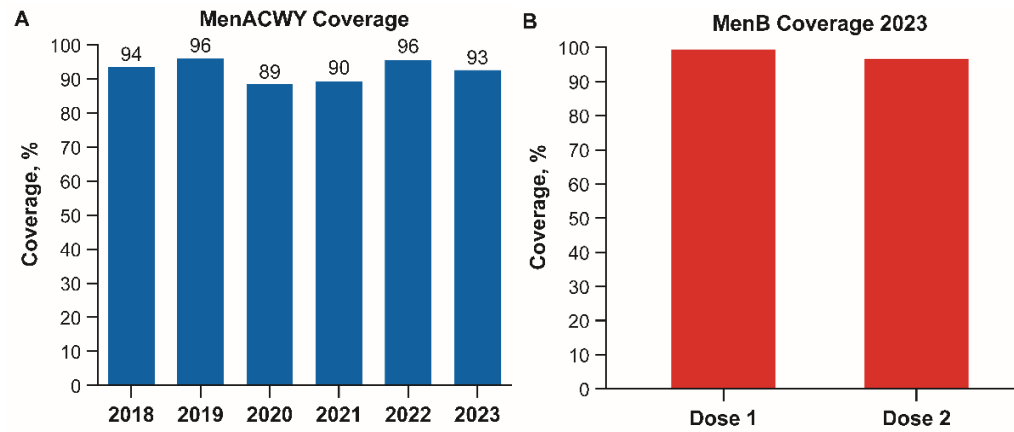

Supplement: Supplementary file 1 [file vaccines-13-01079-s001.zip › vaccines-3837401_Supplementary Figure S2.pdf]
